# Inhibitory Effect of Ophthalmic Solutions against SARS-CoV-2: A Preventive Action to Block the Viral Transmission?

**DOI:** 10.3390/microorganisms9081550

**Published:** 2021-07-21

**Authors:** Francesco Petrillo, Annalisa Chianese, Maddalena De Bernardo, Carla Zannella, Marilena Galdiero, Michele Reibaldi, Teresio Avitabile, Giovanni Boccia, Massimiliano Galdiero, Nicola Rosa, Gianluigi Franci

**Affiliations:** 1 Department of Ophthalmology, University of Catania, 95123 Catania, Italy; francescopetrillo09@gmail.com (F.P.); t.avitabile@unict.it (T.A.); 2 Department of Experimental Medicine, University of Campania “Luigi Vanvitelli”, 80138 Naples, Italy; annalisa.chianese@unicampania.it (A.C.); carla.zannella@unicampania.it (C.Z.); marilena.galdiero@unicampania.it (M.G.); massimiliano.galdiero@unicampania.it (M.G.); 3 Department of Medicine, Surgery and Dentistry “Scuola Medica Salernitana”, University of Salerno, 84081 Baronissi, Italy; mdebernardo@unisa.it (M.D.B.); gboccia@unisa.it (G.B.); 4 Department of Surgical Science, Eye Clinic, University of Turin, 10124 Turin, Italy; mreibaldi@libero.it

**Keywords:** ophthalmic solutions, ocular transmission, antiviral activity, SARS-CoV-2, ocular surface

## Abstract

In 2020, a global pandemic was declared following the spread of SARS-CoV-2, the pathogen responsible for COVID-19. The risk of infection is high due to the ease of transmission, which can occur orally, through droplets, or via contact with contaminated surfaces and objects. It has also been demonstrated that the ocular surface can constitute a transmission route, especially in hospital settings, where health care workers can become a dangerous source of infection. In order to increase prevention and reduce the spread of the virus on the ocular surface, the antiviral activity of already-marketed eye drops against SARS-CoV-2 was evaluated. Iodim, Ozodrop, Septavis, and Dropsept were tested against SARS-CoV-2 in plaque-assay experiments at different stimulation times. Furthermore, the expression levels of early and late genes were evaluated through molecular assays. Results indicated that three of the four ophthalmic solutions showed a considerable dose-dependent inhibition of viral replication, highlighting their use as potential antiviral drugs against SARS-CoV-2 and preventing other ocular infections.

## 1. Introduction

Severe acute respiratory syndrome coronavirus 2 (SARS-CoV-2) is the novel coronavirus responsible for coronavirus disease 2019 (COVID-19). It belongs to the Betacoronavirus genus of *Coronaviridae* family, which is the same as that of both severe acute respiratory syndrome 1 (SARS-CoV-1) and Middle East respiratory syndrome (MERS), the two coronaviruses that caused epidemics in 2003 and 2014, respectively. SARS-CoV-2 was first reported in China in December 2019, and then spread widely all around the world, leading the World Health Organization (WHO) to declare a global pandemic in March 2020 [1,2,3]. To date, there have been almost 168 million cases worldwide and the death toll is expected to be around 3.5 million people. SARS-CoV-2 appears to be less lethal than SARS-CoV-1 or MERS, although its transmissibility is higher and occurs before, during, and after the acute clinical phase of illness. It spreads primarily through respiratory droplets—released while talking, coughing, and sneezing—or by direct or indirect contact of nasal, oral, and ocular mucosa with contaminated surfaces [4,5,6]. Before the entire population receives the vaccine and in the absence of a specific therapy against the virus, it is vital to restrict its spread and look for new antiviral therapies. There are some measures suggested to prevent the virus from spreading [7,8,9]: first of all, social distancing; secondly, the use of Personal Protection Equipment (PPE)—masks, eyewear, and visors—in order to create a physical barrier to viral transmission; finally, hand-washing and disinfection of surfaces in order to chemically remove the virus and avoid its transmission to new hosts [4,10]. 

To date, the possibility of spreading through the ocular route has been widely discussed [11,12]. The presence of the virus on the ocular surface has been demonstrated using PCR in various studies and ophthalmologists are playing an essential role during this pandemic [13,14,15,16,17,18]. Three possible mechanisms of transmission through the ocular route have been suggested: (i) the ocular surface could represent the primary site of infection and replication of the virus; (ii) the virus could move from the ocular surface to the nasal and oral cavity via the nasolacrimal duct; and (iii) infection could occur at the lacrimal gland and be followed by hematic dissemination [17,19,20,21,22]. 

Several studies have demonstrated the efficacy of antiseptic solution to inactivate SARS-CoV-2 in vitro: J.S. Pelletier et al. demonstrated the importance of Povidone-Iodine (PVP-I) for use in the nasal passages, nasopharynx, and oral cavities [5,23,24,25]. S. Frank et al. demonstrated the complete inactivation of SARS-CoV-2 using concentrations of the nasal antiseptic PVP-I as low as 0.5% after 15 s of contact [26,27,28,29]. Hydrogen peroxide, at the recommended oral rinse concentrations of 1.5% and 3.0%, was minimally effective as a viricidal agent after contact times not less than 30 s [30,31,32,33]. The use of antiseptic solution could be fundamental to reducing viral infection, especially for specific categories of people exposed to this route of transmission, such as health care workers, among which the incidence of ocular infections is higher. For this reason, the aim of our study is to evaluate the efficacy of commercial ophthalmic solutions (Iodim, Ozodrop, Dropsept, and Septavis) to inactivate virus replication and prevent its transmission. 

## 2. Materials and Methods

### 2.1. Test Compounds

Ozodrop, Dropsept, Septavis, and Iodim are eye drops that are already marketed and used as ophthalmic solutions for the protection of eyes. Lipozoneye (Ozodrop, FB Vision, Ascoli Piceno, Italy) is a solution made up of ozonated vegetable oil, hydroxypropyl methylcellulose, liposomes, boric acid, sodium tetraborate, disodium edetate sodium, PHMG, and deionized water. Vitamin E TPGS (Dropsept, IROMED group s.r.l., Roma, Italy) contains vitamin E TPGS, dibasic sodium phosphate, monobasic sodium phosphate, sodium chloride, chlorhexidine digluconate, and purified water. Sodium hypochlorite (Septavis, MEDIVIS, Catania, Italy) is a mixture of sodium hypochlorite, sodium chloride, sodium phosphate, hydrochloric acid, and water. Iodine (Iodim, MEDIVIS, Catania, Italy) is composed of medium chain triglycerides, sodium hyaluronate, glycerol, vitamin E TPGS, potassium citrate, sodium chloride, citric acid monohydrate, PVP-I 0.6%, and pure water.

### 2.2. Cytotoxic Activity

To evaluate the cytotoxic activity of ophthalmic solutions, a 3-(4,5-dimethylthiazol-2-yl)-2,5-diphenyltetrazolium bromide (MTT) assay was performed. The day before treatment, 2 × 10^4^ Vero cells/well were seeded in a 96-well plate. Subsequently, the eye drops, positive control (medium and cells), and negative control (DMSO) were added to the cell monolayer. Different volumes of solutions (100–50–25–12.5 µL) were evaluated at different times of stimulation (15 s, 30 s, 1 min, 10 min, 30 min, 1 h, 2 h). After that, MTT solution was added to the cells and they were incubated for 3 h (as reported in the datasheet). A total of 100 µL of DMSO was added to each well to solubilize the formazan and the viability was assessed at 570 nm through a Bio-Rad microplate reader (Bio-Rad Laboratories, Hercules, CA, USA).

### 2.3. Viral Strains and Cell Culture Conditions

SARS-CoV-2 (strain VR PV10734, kindly donated by the Lazzaro Spallanzani Hospital of Rome, Italy) was propagated on Vero cells, epithelial kidney cells of *Cercopithecus aethiops* (ATCC CCL-81) that are very susceptible to SARS-CoV-2 infection. The culture medium used for cell growth was Dulbecco’s Modified Eagle Medium (DMEM) with 4.5 g/L glucose, along with 2 mM L-Glutamine, 100 IU/mL penicillin-streptomycin solution, and 10% Fetal Bovine Serum (FBS). All materials used for cell culture were acquired from Thermo Fisher (Waltham, MA, USA).

### 2.4. Plaque Reduction Assays

To evaluate the effect of the eye drops on SARS-CoV-2 infectivity, four different plaque reduction assays were performed [34]: (a) co-treatment, in which each eye-drop and virus at 0.01 multiplicity of infection (MOI) were simultaneously incubated on the cell monolayer (2.8 × 10^5^ cells in each well); (b) virus pre-treatment, in which each eye-drop was first put together with the virus at 0.1 MOI for different times of stimulation, as shown below, and the mixture was then diluted on the target cells (2.8 × 10^5^ cells for well) for 2 h (viral absorption time); (c) cell pre-treatment, in which 2.8 × 10^5^ cells were pre-treated with each ophthalmic solution and then infected with the virus at 0.01 MOI; and d) post treatment, an assay in which cells (2.8 × 10^5^) were infected with the virus (0.01 MOI) for 2 h and then incubated with each compound at several time points of the stimulation (15 s, 30 s, 1 min, 5 min, 10 min, 15 min, 30 min, 1 h, and 2 h). For all the experiments, different volumes of compounds (12.5, 25, 50, and 100 µL) were used for each time point. At the end of each treatment, the cell monolayer was washed with Phosphate Buffered Saline (PBS) 1X and incubated for 48 h in DMEM supplemented with carboxymethylcellulose (CMC) 5%. After 2 days, the cells were fixed and stained with 0.5% crystal violet, and the plaques were counted. The experiments were performed in triplicate. Ivermectin, an anti-parasitic agent, was used as a positive control (CTR+). The percentage of viral inhibition was calculated compared to the untreated SARS-CoV-2 control (CTR-) as follows:

% Viral inhibition = [100 − (plaques counted in the test sample)/(plaques counted in the negative control)] × 100.

### 2.5. Real-Time PCR

The antiviral potential of eye drops was also investigated through molecular tests. The virus pre-treatment assay described above was performed, with identical conditions. After 24 and 48 h post-infection, the total RNA was isolated using TRIzol reagent (Thermo Fisher, Waltham, MA, USA) and quantified through its absorbance at 260/280 nm (NanoDrop 2000, Thermo Fisher Scientific, Waltham, MA, USA). Then, 1 µg of total RNA was converted to cDNA by 5× All-In-One RT Master Mix (Applied Biological Materials, Richmond, VA, Canada). Quantitative polymerase chain reaction was run in triplicate using a CFX Thermal Cycler (Bio-Rad, Hercules, CA, USA). A total of 2 µL of cDNA was amplified using BrightGreen 2× qPCR MasterMix-No Dye (Applied Biological Materials, Richmond, VA, Canada) and 0.1 µM of primer. The relative target threshold cycle (Ct) values of the spike protein (S) and nucleocapsid protein (N) were normalized to Glyceraldehyde 3-phosphate dehydrogenase (GAPDH), used as a housekeeping gene. The mRNA levels of cells treated with the eye drops were expressed using the 2-ΔΔCt method. Thermocycler conditions for the real-time PCR were as follows: 95 °C for 10 min and 95 °C for 15 s, plus 60 °C for 1 min for 40 cycles. The primers used for real-time PCR are as follows: S Forward (5′-AGGTTGATCACAGGCAGACT-3′), S Reverse (5′-GCTGACTGAGGGAAGGAC-3′), N Forward (5′-GGGGAACTTCTCCTGCTAGAAT-3′), N Reverse (5′-CAGACATTTTGCTCTCAAGCTG-3′), GAPDH Forward (5′-CCTTTCATT-GAGCTCCAT-3′) and GAPDH Reverse (5′-CGTACATGGGAGCGTC-3′).

### 2.6. Statistical Analysis

All tests were performed in triplicate and expressed as mean ± Standard Deviation (SD) calculated by GraphPad Prism (version 5). Statistical differences were evaluated via two-way ANOVA followed by a Bonferroni post hoc test; a value of *p*  ≤ 0.05 was considered significant.

## 3. Results 

### 3.1. Cytotoxic Activity

**Figure 1:**
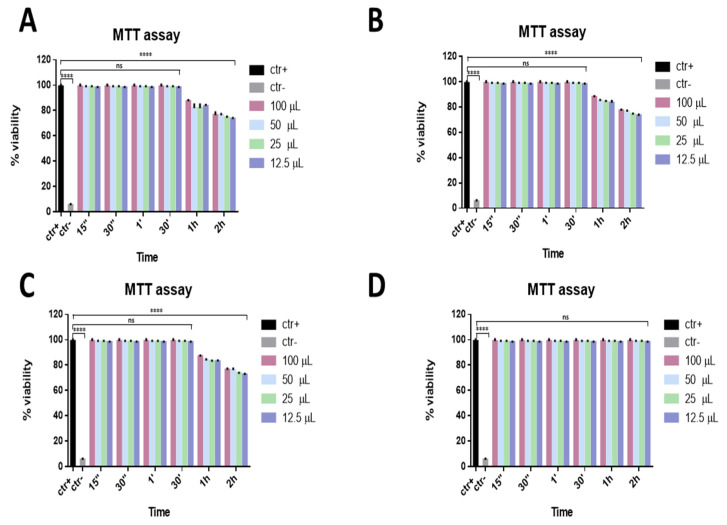
Viability assay. Cytotoxicity was assessed via MTT assay after different stimulation times. Vero cells were exposed to different volumes of Iodim (**A**), Ozodrop (**B**), Dropsept (**C**), and Septavis (**D**) for 15 s, 30 s, 1 min, 30 min, 1 h, and 2 h. **** *p* < 0.0001; ns: non-significant.

In order to evaluate the potential cytotoxicity of ophthalmic solutions, an MTT assay was performed. This is shown in Figure 1, reporting the percentage of viability of ophthalmic solutions on Vero cells. Data showed that a considerable reduction in viability was not observed for all the compounds. In detail, setting 50% cell viability as a threshold line (CC50), Iodim, Ozodrop, and Dropsept showed the highest viability from 15 s to 30 min, and about 70–80% of the viability was observed at the other two times (1 and 2 h). In contrast, only Septavis exhibited 100% cell viability for all the times and volumes analyzed.

### 3.2. Antiviral Activity against SARS-CoV-2

**Figure 2:**
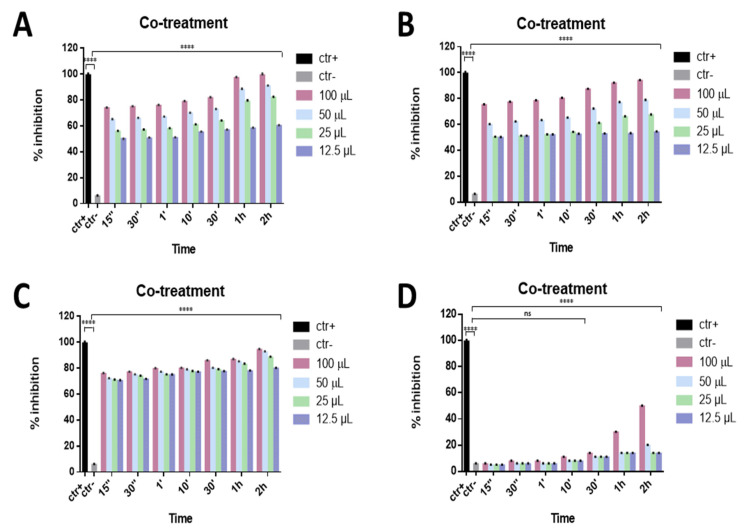
Co-treatment assay. Antiviral activity against SARS-CoV-2 of eye drops at different times of stimulation. Iodim (**A**), Ozodrop (**B**), Dropsept (**C**), and Septavis (**D**) were incubated simultaneously with SARS-CoV-2 on the cell culture for the several time points. Iodim, Ozodrop, and Dropsept inhibited the early stages of infection. On the contrary, Septavis was not able to block the viral replication. **** *p* < 0.0001; ns: non-significant.

The antiviral effect of the eye drops was evaluated against SARS-CoV-2, since many studies have reported that the viral infection can also be characterized by severe ocular disease. The ability to interfere with SARS-CoV-2 life cycle through a co-treatment assay was preliminarily investigated for all compounds. The virus and each eye drop at the indicated volumes were incubated together on the cells for different times, from 2 h to 15 s, at 37 °C. Setting 50% viral inhibition as the threshold line, Septavis was the least effective against SARS-CoV-2, while the other three eye drops showed a considerable dose-dependent inhibition of the viral replication. They showed similar inhibitory activity at the highest time point: Iodim, Ozodrop, and Dropsept exhibited a half-maximal inhibitory concentration (IC50) at 12.5 µL until 15 s, and they were able to totally inhibit SARS-CoV-2 infection at the higher volume of 50 µL (Figure 2). 

**Figure 3:**
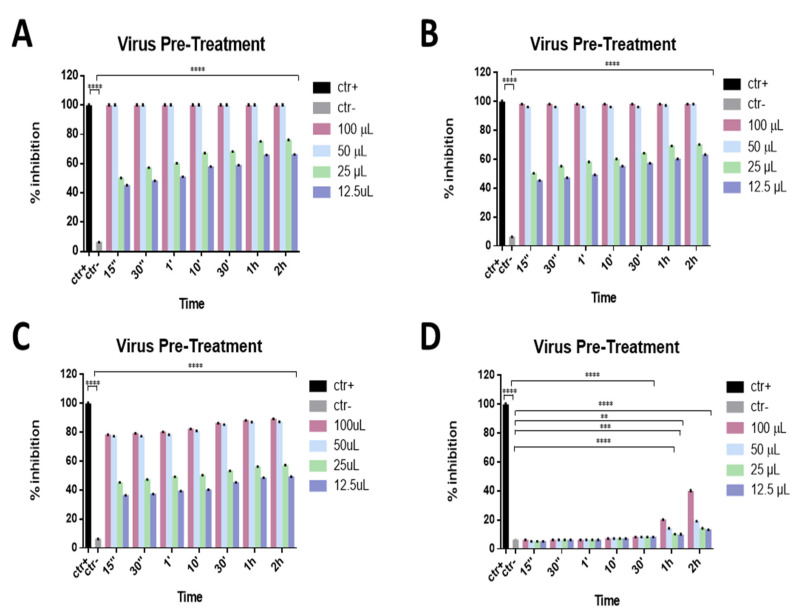
Virus pre-treatment assay. Antiviral activity against SARS-CoV-2 of eye drops at different times of stimulation. Iodim (**A**), Ozodrop (**B**), Dropsept (**C**), and Septavis (**D**) were incubated first with SARS-CoV-2 and then the mixture was titrated on Vero cells. Iodim, Ozodrop, and Dropsept inhibited the early stages of infection, while Septavis was not able to block the viral replication. ** *p* < 0.001; *** *p* = 0.0001; **** *p* < 0.0001; ns: non-significant.

To evaluate the mechanism of action of these compounds in detail, a virus pre-treatment test was also performed. In this approach, the virus was incubated with the eye drops for 1 h, 30 min, 10 min, 1 min, 30 s, and 15 s at 37 °C; the mixture was then diluted and added to the Vero cell monolayer. As reported in Figure 3A,B, only two eye drops (Iodim and Ozodrop) showed strong virucidal activity against SARS-CoV-2. In particular, “Iodim” and “Ozodrop” exhibited high antiviral activity with a 98% inhibition of virus plaques at 50 μL until 15 s, while “Dropsept” showed a 77% inhibition against SARS-CoV-2 at 50 µL until 15 s (Figure 3C). Finally, “Septavis” did not exhibit any activity in the same conditions (Figure 3D).

**Figure 4:**
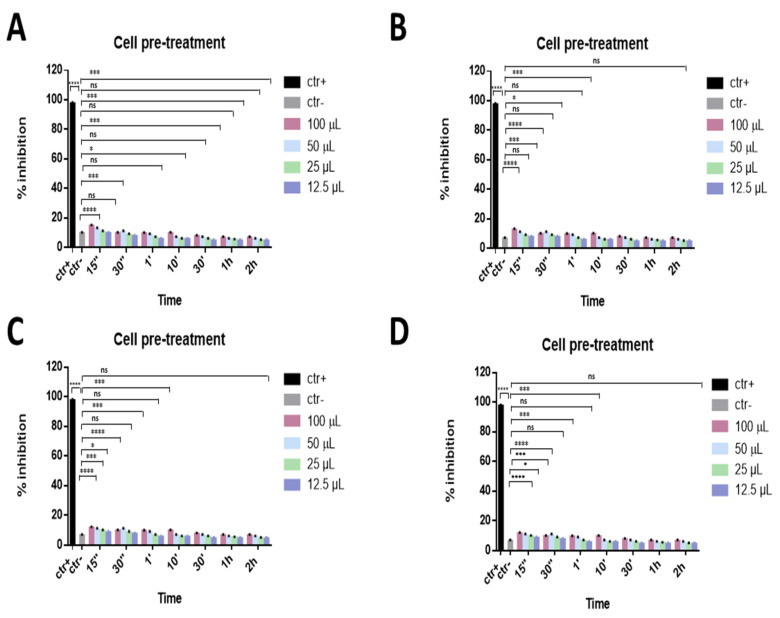
Cell pre-treatment assay. Antiviral activity against SARS-CoV-2 of eye drops at different times of stimulation. Iodim (**A**), Ozodrop (**B**), Dropsept (**C**), and Septavis (**D**) were first incubated on the cell monolayer prior to infect them. Iodim, Ozodrop, Dropsept, and Septavis were not able to interact with the cellular surface. * *p* = 0.001; *** *p* = 0.0001; **** *p* < 0.0001; ns: non-significant.

Furthermore, a cell pre-treatment assay was carried out to evaluate if compounds could also act on the cell surface, perhaps by interacting with cellular receptors and blocking the virus–cell fusion. In this case, Vero cell monolayers were precooled and incubated with the eye drops for different times (1 h, 30 min, 10 min, 1 min, 30 s, and 15 s) at 4 °C; afterwards, cell monolayers were infected with SARS-CoV-2. As reported in Figure 4, the data indicated that compounds were not involved in any cell surface mechanism of SARS-CoV-2 infection.

**Figure 5:**
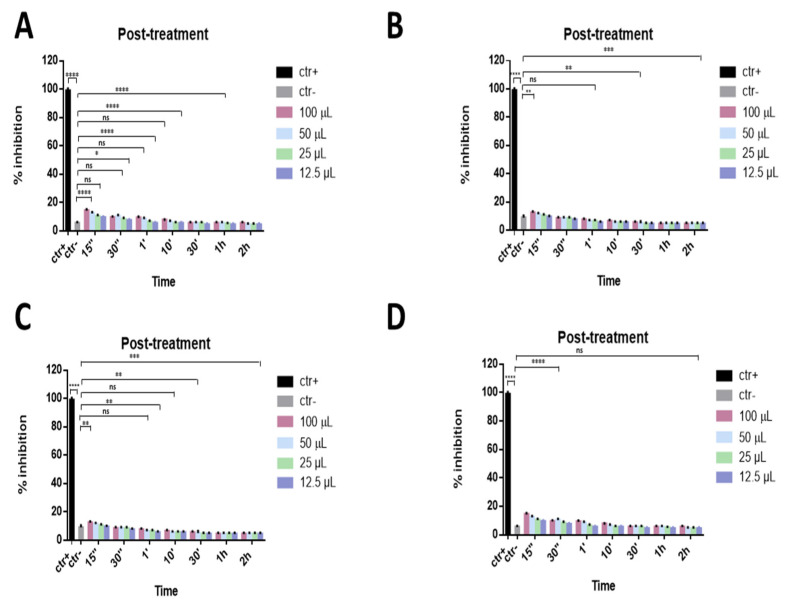
Post-treatment assay. Antiviral activity against SARS-CoV-2 of eye drops at different times of stimulation. Cells were first infected with SARS-CoV-2 and then treated with Iodim (**A**), Ozodrop (**B**), Dropsept (**C**), and Septavis (**D**). Iodim, Ozodrop, Dropsept, and Septavis were not able to act on the viral replication mechanism. * *p* = 0.001; ** *p* < 0.001; *** *p* = 0.0001; **** *p* < 0.0001; ns: non-significant.

Finally, to investigate if the ophthalmic solutions act on viral replication, a post-treatment assay was performed. In this approach, different volumes of eye drops were added to the cell monolayer for different times after infection with SARS-CoV-2. All samples did not act inside the cell by interfering with the viral replication phase, as shown in Figure 5.

### 3.3. Real-Time PCR

To determine whether treatment with the eye drops could interfere with active viral replication, real-time PCR was carried out.

First, the different levels of total viral DNA were analyzed following treatment with eye drops compared to untreated SARS-CoV-2 at 24 and 48 h post-infection at different times of stimulation (15 s, 30 s, 1 min, 10 min, 30 min, 1 h, 2 h). 

**Figure 6:**
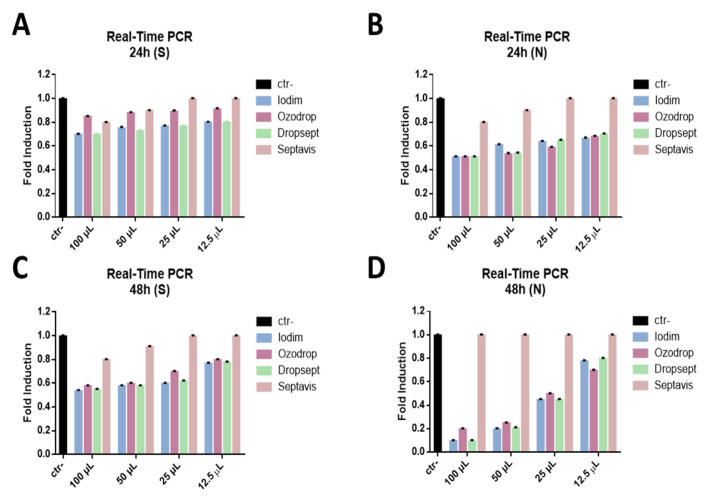
Molecular assay. Real-time PCR was performed to evaluate the effect of eye drops on the viral gene expression. Iodim (**A**), Ozodrop (**B**), Dropsept (**C**), and Septavis (**D**) were tested at different volumes (from 100 to 12.5 uL), for 2 h stimulation at 24 and 48 h p.i. The expression of the spike glycoprotein (S) (**A**,**B**) and nucleocapsid protein (N) (**C**,**D**) was analyzed. Ctr- refers to infected but not treated cells.

The expression of the nucleocapsid protein (N), which is an early gene, and the spike glycoprotein (S), involved in the entry of the virus into the cell, was investigated. The results showed that all compounds blocked the expression of N and reduced the S expression at all stimulation times analyzed, from 15 s to 2 h (Figure 6). 

These data indicate that the eye drops have an early action, functioning outside the cell, preventing the virus from entering the cell, thus blocking the virus directly.

## 4. Discussion

In 2020, a global pandemic was declared following the spread of SARS-CoV-2, the pathogen responsible for COVID-19 [35]. The risk of infection is high due to the ease of transmission, which can occur orally, through droplets, or via contact with contaminated surfaces and objects. It has also been demonstrated that the ocular surface can also constitute a transmission route, especially in hospital settings, where health care workers can become a dangerous source of infection. 

The global pandemic caused by the marked spread of SARS-CoV-2 has caused an increase in the mortality rate worldwide. It is therefore of fundamental importance to reduce the infection and increase prevention to limit viral spread. 

Our goal was to provide antiseptic solutions able to limit SARS-CoV-2 transmission through the ocular surface. We also aimed to assess which solution tested was the most successful in reducing viral replication. Four commercial eye drops were tested.

As reported in the literature, several studies have been conducted to evaluate the antimicrobial activity of eye drops, both in vitro and in vivo. Celenza et al. examined the antifungal activity of ozone-based eye drops on different strains of Candida, finding a significant inhibition [36,37,38,39,40]. 

The bactericidal activity of Ozodrop was also evaluated in vivo in patients (both animals and humans) with ocular infections caused by Gram-positive and Gram-negative bacteria (such as *S. aureus* and *P. aeruginosa*) [41,42,43,44,45]. Ozodrop contains ozonated oils, the antiviral activity of which was recently described, finding that it was due to the oxidation of specific viral receptors [46]. Furthermore, for Iodim the antibacterial and antifungal activities were examined at different times, showing the inhibition of bacterial and fungal growth after 5 min and 24 h of incubation, respectively. PVP-I, present in Iodim, has been largely reported on for its antiviral potential against human immunodeficiency virus (HIV) type 1 and also human and avian influenza A viruses [47]. In detail, Sriwilaijaroen et al. [48] demonstrated that 1.56 mg/mL of PVP-I reduced the infection of 8 human and 5 avian influenza A strains, including H1N1, H3N2, H5N3, and H9N2. This inhibitory action ranged from 23.0 to 97.5% and has been ascribed to the effect on hemagglutination and sialidase activities. Very recently, Singh et al. investigated the PVP-I activity against two RNA viruses, Zika and Chikungunya [49]. It was demonstrated that PVP-I at 0.01% was very effective in reducing the viral replication in corneal and retinal cells. As our active eye drops, it showed a very strong virucidal action after only one minute of incubation, without causing a relevant toxicity on the treated cells. Caruso C., et al. demonstrated the efficacy of Vitamin E TPGS (Dropsept) for treating *Acanthamoeba* *keratitis* (AK), a rare infection of the cornea caused by the ubiquitous protozoan [50]. Another component of Dropsept, the chlorhexidine-digluconate, has been known to possess a relevant antiviral action against several enveloped viruses since 1990 [51].

The antimicrobial activity of Septavis is not known and has not been reported in the literature [45,52,53]. Comparing these data with the antiviral activity evaluated in this study, the efficacy of the eye drops is also confirmed at the antiviral level with about 50% inhibition at the minimum time tested (15 s). We hypothesized that ophthalmic solutions could act and prevent the initial phases of SARS-CoV-2 infection, such as the viral attachment and entry to the target cell, thus interfering with the downstream infection process.

These data were also confirmed by carrying out molecular tests, in which the compounds inhibited the expression of N proteins and reduced the expression of S proteins.

Together, these findings showed that the eye drops may act on virus attachment to the host cell by directly blocking the virus particle as a virucide and deactivating it irreversibly. Following the remarkable inhibitory effect shown by the eye drops against the virus, it is possible to deduce that these solutions could represent a preventive resource for ocular infections. 

## Data Availability

Not applicable.
